# *DIALS*: implementation and evaluation of a new integration package

**DOI:** 10.1107/S2059798317017235

**Published:** 2018-02-01

**Authors:** Graeme Winter, David G. Waterman, James M. Parkhurst, Aaron S. Brewster, Richard J. Gildea, Markus Gerstel, Luis Fuentes-Montero, Melanie Vollmar, Tara Michels-Clark, Iris D. Young, Nicholas K. Sauter, Gwyndaf Evans

**Affiliations:** a Diamond Light Source Ltd, Harwell Science and Innovation Campus, Didcot OX11 0DE, England; b Lawrence Berkeley National Laboratory, 1 Cyclotron Road, Berkeley, CA 94720, USA; c STFC Rutherford Appleton Laboratory, Didcot OX11 0FA, England; d CCP4, Research Complex at Harwell, Rutherford Appleton Laboratory, Didcot OX11 0FA, England; e Laboratory of Molecular Biology, Francis Crick Avenue, Cambridge CB2 0QH, England

**Keywords:** X-ray diffraction, data processing, methods development, *DIALS*

## Abstract

The *DIALS* project is a collaboration between Diamond Light Source, Lawrence Berkeley National Laboratory and CCP4 to develop a new software suite for the analysis of crystallographic X-ray diffraction data, initially encompassing spot finding, indexing, refinement and integration. The design, core algorithms and structure of the software are introduced, alongside results from the analysis of data from biological and chemical crystallography experiments.

## Abstract 2

A new X-ray diffraction data-analysis package is presented with a description of the algorithms and examples of its application to biological and chemical crystallography.

## 1. Introduction   

X-ray crystallography is the dominant method for the determination of the atomic structure of biological macromolecules. Macromolecular crystallography (MX) has evolved over decades into an essentially routine method for the majority of structures being investigated. Incremental improvements in detector technology, X-ray sources, beamline instrumentation (both in optics and endstation) and automation of sample handling have contributed to the success of the method. The overwhelming majority of diffraction data resulting in PDB depositions over the last 2–3 decades have been analysed using just four programs: *XDS* (Kabsch, 2010*b* ▸), *MOSFLM* (Leslie, 2006 ▸), *HKL*-2000/*DENZO* (Otwinowski & Minor, 1997 ▸) and d*TREK (Pflugrath, 1999 ▸). For chemical crystallo­graphy, *SAINT* (Bruker AXS Inc., Madison, Wisconsin, USA) and *EVAL* (Duisenberg *et al.*, 2003 ▸; Schreurs *et al.*, 2010 ▸) as well as d*TREK are in common use. Significant effort by a relatively small number of developers over this time has been critical to producing the diffraction-intensity data sets that are the raw material of structure determination.

In more recent years there has been a step change in MX throughput, driven principally by the availability of new X-ray sources and data-collection methodologies (Emma *et al.*, 2010 ▸; Ishikawa *et al.*, 2012 ▸; White *et al.*, 2012 ▸; Gati *et al.*, 2014 ▸; Stellato *et al.*, 2014 ▸; Sierra *et al.*, 2016 ▸; Fuller *et al.*, 2017 ▸), high-frame-rate pixel-array detectors (Henrich *et al.*, 2009 ▸), fast sample exchange (Russi *et al.*, 2016 ▸) and automated data analysis (Winter, 2010 ▸; Winter & McAuley, 2011 ▸; Vonrhein *et al.*, 2011 ▸). This allows larger numbers of smaller samples to be used, with correspondingly more challenging data. New algorithms and approaches to data analysis are therefore required to address the novel approaches to the measurement of diffraction data sets. The initial focus of the development of *DIALS* (*Diffraction Integration for Advanced Light Sources*) has been on the processing of data from pixel-array detectors, although other technologies such as CCDs are also supported.

To develop new algorithms, it is necessary to have the infrastructure of an existing software package to support them. A suitably extensible open-source package did not exist, and the *DIALS* project was initiated to provide this platform. The project aims to deliver (i) a framework for the implementation of novel algorithms for the analysis of X-ray diffraction data; (ii) a toolbox of algorithms within this framework; and (iii) a collection of user-friendly tools to present the structural biologist with an interface to the analysis of rotation data sets collected at synchrotron sources, as well as still-shot diffraction data collected at both synchrotron and X-ray free-electron laser sources.

*DIALS* is built upon the *cctbx* library (*Computational Crystallography Toolbox*; Grosse-Kunstleve *et al.*, 2002 ▸) and benefits from a substantial foundation of crystallographic and mathematical code, a robust build mechanism and a development platform using hybrid Python/C++ (Abrahams & Grosse-Kunstleve, 2003 ▸).

Finally, while the main focus of *DIALS* to date has been the analysis of MX data, the aforementioned developments in instrumentation also apply to chemical crystallography (CX). Since the analysis is mathematically identical, *DIALS* has also targeted data from this field, bringing a new set of challenges. This has the benefit of ensuring mathematical rigour and flexibility in the future, since assumptions which may be appropriate for MX may be challenged by CX and *vice versa*.

## 2. Design overview   

The core aim of *DIALS* is to allow the development of a wide range of algorithms within a single framework. The workflow of *DIALS* was decomposed into a number of discrete tasks exchanging information *via* data files, in a similar manner to *XDS* and d*TREK. During the early stages of development, this allowed the implementation of standalone algorithms based on the results of other software such as *MOSFLM* (Leslie, 2006 ▸) and *XDS* (Kabsch, 2010*a* ▸). This decomposition also makes testing of the *DIALS* software more straightforward and facilitates its inclusion within automated data-analysis systems.

**Figure 1:**
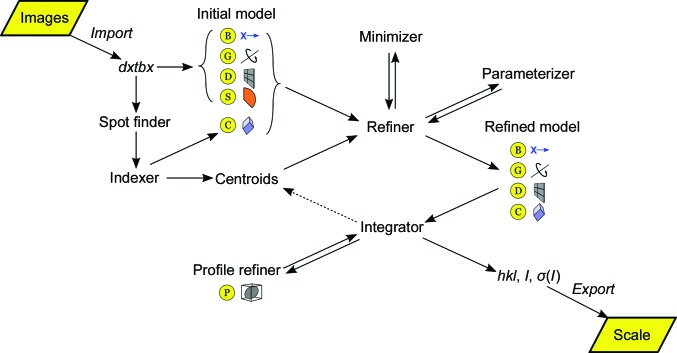
Flow diagram illustrating the scope and workflow of *DIALS*. The experiment is represented by a set of abstract models describing the parameters of the X-ray beam (B), goniometer (which incorporates the description of the goniometer hardware; G), imaging detector (D), scan (which includes goniometer settings for a given sequence of images and exposure times; S), crystal (C) and Bragg spot profile (P). The reflection data are passed from one step to the next as a list, with the properties of the reflections extended as processing proceeds.

The workflow of *DIALS*, as expressed in Fig. 1 ▸, emphasizes the abstract procedure for processing X-ray diffraction data and reflects the division of tasks as described previously (Bricogne, 1986*b* ▸; Pflugrath, 1999 ▸; Winter, 2010 ▸). Beginning with the handling of the X-ray diffraction data in the Diffraction Experiment Toolbox *dxtbx* (Parkhurst *et al.*, 2014 ▸), abstract interfaces have been used at key points to ensure that future algorithms may be implemented within *DIALS* with minimum disruption.

### 2.1. Data handling   

The *dxtbx* offers a general, user-extensible interface for the reading of X-ray diffraction data and provides abstract models in C++ and Python to describe the derived experimental geometry. For example, within the *dxtbx* the geometry of a detector is expressed as a collection of abstract planes, each of which has a per-pixel mapping from the position on the surface to the pixel coordinates in the image. This mapping may be used to correct for static effects such as module position or CCD taper corrections, or for dynamic effects such as parallax correction in direct-conversion detectors (described in more detail in *Appendix A* [App appa]). The interface exposed to the rest of the *DIALS* software is consistent, regardless of the underlying detector implementation, and has been used to treat data from new and complex detectors such as the CSPAD (Hart *et al.*, 2012 ▸) used for XFEL data collection at the Linac Coherent Light Source (Herrmann *et al.*, 2014 ▸; Brewster *et al.*, 2016 ▸), the DECTRIS PILATUS 12M used for long-wavelength data collection (Wagner *et al.*, 2016 ▸) at Diamond Light Source beamline I23, and HDF5-format (https://www.hdfgroup.org/HDF5) DECTRIS EIGER data sets (Casanas *et al.*, 2016 ▸).

### 2.2. Data structures   

The *DIALS* framework defines two major data structures for data persistence and transfer between algorithms and applications. The reflection table is a column-centric database of reflection properties with methods specialized for performing data-processing operations on a set of reflections. The experiment list encodes the experimental geometry and crystal properties. Each experiment has exactly one beam, detector and crystal model, with an optional goniometer and scan model; an experiment list is a collection of these. Models may be shared between experiments; for example, for data collected from multiple crystals, the beam, detector and goniometer models can be shared between all of the experiments, with the crystal and scan models differing for each. The relationships between different data collections can be used to provide additional information in, for example, joint refinement against multiple data sets whose sets of experimental models intersect. This has been detailed in Waterman *et al.* (2016 ▸).

In the command-line *DIALS* programs the input and output are defined as reflection tables and experiment lists, and in most cases the input and output are one of each, with additional parameters being passed as keyword=value pairs.

## 3. Implementation   

The initial effort within the *DIALS* project has focused on delivering the key components of a complete integration package; namely, spot finding, indexing, refinement and integration, *i.e.* to take as input X-ray diffraction data from an area detector and output background-subtracted integrated intensities and associated error estimates. *DIALS* applications are implemented using the hybrid programming model of *cctbx*. Computationally demanding algorithms are implemented in C++, with Python wrappers to allow flexible high-level application development. This facilitates the construction of multiple user interfaces to the core algorithms of *DIALS*. For steps such as integration, where alternative algorithms are envisaged, a plugin system has been developed to allow run-time extension of the *DIALS* software, providing a convenient means for the development of new algorithms.

### 3.1. Algorithms: spot finding   

The default spot-finding algorithm in *DIALS* performs a pixel thresholding process followed by the determination of connected regions (in two dimensions for still shots or three dimensions for rotation data) and size, centre of mass and total intensity estimation. The resulting spot list is then filtered based on user criteria, *e.g.* the minimum and maximum number of pixels in a spot.

The default method for identifying strong pixels is based on the method used by *XDS*: the local mean, μ, and variance, σ^2^, are calculated for each pixel (over the region around the pixel defined by the kernel size) in each image and subsequently the local index of dispersion
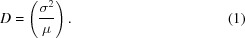
For a detector with insignificant point-spread and gain *G*, a value of *D* ≃ *G* is expected for the background, with *G* being unity for a photon-counting detector. The appropriate gain for integrating detectors is normally set by the relevant *dxtbx* format class, but if required the value can be modified for spot finding. Strong pixels are then identified through three sequential thresholding operations. Firstly, pixels with a value less than a global threshold value (by default set to zero) are discarded. Next, a gain-dependent threshold is applied using the index of dispersion map to identify regions of the image that contain strong pixels. This operation essentially tests for regions of the image whose pixels are not drawn from a single Poisson distribution, *i.e.* not a local flat field. For Poisson-distributed data, the quantity *D*(*N* − 1) is approximately χ^2^ distributed with *N* − 1 degrees of freedom, where *N* is the number of pixels in the region (Frome, 1982 ▸). Therefore, the expected variance in *D*(*N* − 1) is 2(*N* − 1). Pixels are marked as potentially strong if the index of dispersion in a local region around the pixel is greater than a certain number of standard deviations, given by the parameter σ_*b*_, above the expected value,
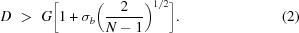
Finally, pixels in these regions are selected as strong if their values *c_i_* are greater than a certain number of standard deviations, given by the parameter σ_*s*_ (assuming a Poisson distribution), above the local mean,
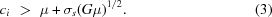
This method will find features on the image, for example Bragg reflections, powder rings and zingers.

For photon-counting detectors the default settings for the global threshold (0) and gain (1) are usually appropriate. For other detectors where these defaults are not correct, appropriate values can be set in the *dxtbx* library as part of the detector model, or manually adjusted during spot finding. Determining appropriate parameters is easily accomplished interactively *via* the image viewer, as described in §[Sec sec5.1]5.1.

With some integration packages the initial spot finding is often limited to a subset of the data for the initial characterization, *i.e.* indexing from a small number of images. Within *DIALS*, the decision was made to globally model the experiment. This decision has a significant effect on spot finding: the recommended usage (although this is not mandatory) is to find spots throughout the entire data set and perform subsequent indexing and refinement using this list of spots or a random subset. The spot list is also used to designate which reflections are used in the construction of reference profiles during integration.

### 3.2. Algorithms: indexing   

Given a list of centroids from a spot-finding routine and a description of the experimental geometry, the primary goal of indexing is to identify a suitable combination of reciprocal-space basis vectors, represented by the **UB** matrix (Busing & Levy, 1967 ▸), that best explains the input list of spot centroids. This task is often complicated by the presence of outliers, either in the form of spuriously identified spot centroids or genuine diffraction spots that do not belong to the principal lattice (for example, ice or salt diffraction or the presence of one or more additional crystal lattices).

Indexing may be algorithmically decomposed into several steps, which are common to most indexing packages, as follows. Given a description of the experimental geometry and a list of spot centroids as described above, the centroids are first mapped to reciprocal space to give a list of reciprocal-lattice positions. This list of positions is then analysed by one of several algorithms to determine a basis set. Once a suitable choice of basis vectors has been made, the resulting orientation matrix is used to assign Miller indices to reciprocal-lattice points, and refinement of the initial crystal parameters and experimental geometry is then performed (see §[Sec sec3.3]3.3).

Analysis of the set of reciprocal-lattice positions to determine the basis may use a variety of algorithms. In *XDS* (Kabsch, 1988*a* ▸) the set of short reciprocal-space difference vectors is calculated to build up a histogram of low-order multiples of lattice vectors, which is analysed to determine a unique basis. Other methods rely on the long-range periodicity of the reciprocal-lattice positions, analysed *via* the Fourier transform, to provide a route for simultaneously determining both the unit-cell and crystal-orientation parameters from a set of observed spot centroids. *DIALS* provides a choice of a one-dimensional (Steller *et al.*, 1997 ▸; Sauter *et al.*, 2004 ▸) or three-dimensional (Bricogne, 1986*a* ▸; Otwinowski & Minor, 1997 ▸; Campbell, 1998 ▸; Otwinowski *et al.*, 2012 ▸) fast Fourier transform (FFT)-based algorithms, or a real-space grid-search method (Gildea *et al.*, 2014 ▸), although the latter requires prior knowledge of the unit-cell parameters.

After successful identification and refinement of a single lattice, if a significant number of unindexed reflections remain then identification of further lattices may be attempted on the remaining unindexed reflections, as described by Gildea *et al.* (2014 ▸).

Unless otherwise specified, the above algorithms find the primitive minimum reduced unit cell (Grosse-Kunstleve *et al.*, 2004 ▸), making no attempt to derive the metric symmetry of the lattice at this point. Once refinement of the crystal parameters and experimental geometry in a triclinic cell has been completed, the Bravais lattice may be determined by applying appropriate constraints on the unit-cell parameters according to each compatible Bravais setting (Sauter *et al.*, 2006 ▸) and repeating the refinement with these constraints. In addition, the symmetry observed in the intensity of the found spots may be assessed by computing the correlation coefficient in the spot intensity across the symmetry operations: if the minimum and maximum correlation coefficients are substantially different it may indicate that the lattice is pseudo-symmetric. While the analysis gives a suggestion of the ‘correct’ solution, the final decision is left to the user.

If diffraction from a single crystal has been recorded on multiple sweeps (for example multiple orientations with a multi-axis goniometer) it is straightforward to index all sweeps simultaneously by passing the geometry and strong reflections from each. This was found to be particularly valuable for indexing data from chemical crystallography experiments, ensuring a consistent definition of **UB** for all data.

### 3.3. Algorithms: refinement   

To date, the majority of packages for the integration of X-ray diffraction data have refined the model (unit cell, crystal orientation, detector distance and orientation, and beam direction) within small blocks during the integration process, just prior to integration of that block, to ensure that reflections in that block are well predicted. This process may take the form of positional refinement (Kabsch, 2010*b* ▸) or post-refinement (Rossmann *et al.*, 1979 ▸; Winkler *et al.*, 1979 ▸; Leslie, 2006 ▸). At the end of integration a further global refinement may be performed to give an accurate unit cell for downstream analysis. Within *DIALS* an alternative approach has been taken in which global refinement is performed prior to integration: this can refine a single static model for the sample (a single **UB** matrix representing the crystal unit cell and orientation) or a model that is allowed to vary smoothly throughout the scan. The latter allows systematic changes in orientation, for example owing to goniometer errors and radiation-induced unit-cell changes, whilst still using a global model. The emphasis on a global model stems from two key goals. The first is to determine the best model to fit the data set as a whole. This avoids instabilities, such as those inherent in refining unit-cell parameters for a low-symmetry crystal from a narrow wedge of data (especially cell axes aligned with the incident beam), and reduces correlations between parameters in refinement. The second goal is to allow maximum parallelism in the integration: as the entire experimental model is known *a priori*, in principle every reflection in the data set may then be integrated simultaneously.

In common with other data-processing packages, refinement is performed by minimizing a least-squares target function. In *DIALS*, the residuals of this target function consist of the differences in position between the observed and predicted spot centroids in the *x* and *y* directions on the detector plane and the rotation angle φ. The squared residuals are weighted by the inverse of the estimated variances in centroid positions such that the resulting target function is dimensionless. As it is assumed that reliable profile information will be available only during the integration stage of data processing, no attempt at traditional post-refinement is made at this stage. Therefore, the refinement is limited to the central impacts (Duisenberg *et al.*, 2003 ▸). Nevertheless, the constraint of either a static or a smoothly changing crystal model for the whole scan reduces correlations between crystal and detector parameters, resulting in more reliable refined unit-cell parameters (Waterman *et al.*, 2016 ▸). Refinement based solely on the spot centroids is a simple but effective way to improve the geometric model of the experiment, particularly when the data are fine-sliced (*i.e.* the image width is less than the mosaic spread; Pflugrath, 1999 ▸). A comprehensive discussion of *DIALS* refinement is given by Waterman *et al.* (2016 ▸).

### 3.4. Algorithms: integration   

Integration within *DIALS* is separated into three steps. The first is the determination of the reflection profile, consisting of pixels that are part of the reflection peak (foreground) and those in the background. The second step estimates the background values *under* the peak. Finally, the peak intensity is evaluated *via* summation integration or profile fitting.

#### 3.4.1. Profile parameters   

The process of integrating the individual reflections within *DIALS* begins with the determination of profile model parameters, enabling the classification of pixels into foreground and background for each reflection. At the time of writing, a single model has been implemented based on the method described by Kabsch (2010*a* ▸) that uses a three-dimensional Gaussian description of the reflection in a local reciprocal-space coordinate system defined by two parameters that determine the extent of the reflection on the face of the detector, σ_D_, and over a range of images, σ_M_. These parameters are estimated from the list of indexed strong spots identified previously during spot finding, as described in Kabsch (2010*a* ▸).

#### 3.4.2. Background estimation   

Using the calculated model parameters, image pixel data are read into reflection ‘shoeboxes’ that contain the peak pixels and a substantial border of background pixels surrounding the peak. Before estimating the reflection intensity, the background in the peak region of the reflection needs to be modelled. This is accomplished by using information from nonpeak pixels in the local area of each spot. An important step in the background modelling is to ensure that the estimated background is not contaminated by outlier pixels such as zingers, unmodelled intensity from adjacent reflections, Bragg diffraction from ice, or reflections from a different lattice.

*DIALS* provides a range of outlier-handling methods which can be used with simple constant and linear background models and are particularly appropriate for CCD data where a pedestal has been subtracted. However, since these traditional methods assume that the pixel values are approximately normally distributed, the background estimates that they produce may be biased for low background levels with modern photon-counting detectors, where the counts are Poisson-distributed. Therefore, the default background-modelling algorithm in *DIALS* uses a robust generalized linear model approach, which explicitly assumes that the pixel values are Poisson-distributed. This method is appropriate across the full range of observed background levels, has been shown to be effective even when the average background is below one count per pixel (Parkhurst *et al.*, 2016 ▸), and is particularly suitable for photon-counting detectors.

#### 3.4.3. Intensity evaluation   

Given an estimate for the background under the peak, the simplest integration algorithm is direct summation, where the integrated intensity is obtained as the sum of all background-subtracted pixel values in the peak region. *DIALS* can output the summation intensities of each reflection as either individual partial reflection intensities or as a single value summed across all of the frames on which the reflection is recorded. Error estimates are derived from Poisson statistics as described by Leslie (1999 ▸).

For weak data, fitting the pixel intensities against an empirical reflection profile has been shown to give better estimates of weak reflection intensities than summation integration (Diamond, 1969 ▸). In *DIALS*, profile fitting is performed as described by Kabsch (2010*a* ▸). The image/rotation-space shoebox for each reflection is first transformed into its local reciprocal-space coordinate system, in which the reflection profiles take on a more uniform appearance, allowing their shapes to be modelled more effectively (Kabsch, 1988*b* ▸). In contrast to *XDS*, the reflection data are transformed onto the reciprocal-space grid by computing the overlap of each detector pixel with the transformed grid point using a polygon-clipping algorithm (Sutherland & Hodgman, 1974 ▸). The fractional overlap is then used to determine the number of counts in each pixel that is distributed to each grid point in the transformed grid.

In order to aid parallel execution, blocks of images are integrated independently. The blocks of images are overlapped so that the start of a block is aligned with the centre of the preceding block. This ensures that the majority of reflections are fully recorded within a single block, with a better profile-fitting intensity estimate than reflections split at block boundaries and reassembled after integration. Reference profiles are created from the strong spots at several points across the detector surface for each block of images being integrated. Each strong reflection contributes to its nearest reference profiles using a Gaussian weight derived from its distance to the reference profile, such that reflections halfway between two reference profiles contribute half of their intensity to each reference profile. Once the reference profiles have been created, the intensity is calculated by fitting the transformed profile of each reflection to the nearest reference profile. The profile-fitted intensity and error are calculated as described by Kabsch (2010*a* ▸).

### 3.5. Algorithms: data correction   

The intensities measured on the X-ray diffraction images are modulated by a range of variable effects including the incident beam intensity, the illuminated volume and the absorption within the sample. The intensities of measured reflections are also affected by known, sample-independent factors, including beam polarization, the velocity of the reciprocal-lattice point through the reflecting position (Lorentz correction) and the detector sensitivity.

The variable effects are normally corrected by scaling procedures such as those implemented in *AIMLESS* (Evans & Murshudov, 2013 ▸) and *XDS* (Kabsch, 1988*b* ▸). The known effects may be corrected for in scaling, as in *XDS*, or could be corrected after integration but prior to scaling, as in *MOSFLM* and *AIMLESS*. The Lorentz and polarization corrections are well defined and have been described in detail elsewhere (Kabsch, 1988*b* ▸). Correction for detector-sensitivity variation is an instrument-specific procedure, the details of which vary for different detector types. For pixel-array detectors (Henrich *et al.*, 2009 ▸), one relevant factor is the probability of recording an individual scattered photon. In particular, the sensor has a fixed thickness of, for example, crystalline silicon (typically between 320 µm and 1 mm), giving rise to a specific probability of a photon being absorbed by the sensor, dependent on the wavelength of the photon and the incident angle,
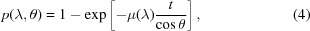
where θ is the angle between the incoming ray and the detector normal, λ is the wavelength of the photon, μ(λ) is the corresponding attenuation coefficient and *t* is the thickness of the sensor (Hülsen *et al.*, 2005 ▸). The intensities should be corrected by a factor of 1/*p* (the oblique incidence correction). For the wavelengths routinely used in MX this correction is modest, typically in the range 1.1–1.25. For the higher energies typically used in CX it may be more substantial (2.0–2.5), as the interaction cross-sections between the photons and the Si atoms are much smaller. The effects are particularly profound when more complex experimental geometries are used, since the correction may not vary uniformly with resolution if the detector is not perpendicular to the beam.

### 3.6. Algorithms: post-integration unit-cell refinement   

The goal of the refinement described earlier (§[Sec sec3.3]3.3) is the accurate prediction of the X-ray diffraction pattern; for downstream analysis, however, a reliable best estimate of the unit cell is critical. After integration, the 2θ angles for individual reflections are very well known and may be used to re-refine the unit-cell parameters directly and also to provide error estimates on the unit-cell parameters. A separate tool is provided for this unit-cell refinement, which shares its underlying framework and models with the general refinement.

## 4. Examples   

The most relevant criteria for judging the integration of X-ray diffraction data are structure solution and refinement using the reduced intensities. Two protein examples follow to illustrate this: (i) structure solution of the leucine-rich repeat protein from *Leptospira interrogans* *via* SAD phasing using a standard SAD strategy for data collection and (ii) a molecular-replacement example (thermolysin) using very weak and high-multiplicity data. A third example, of structure solution and refinement of a small-molecule structure, is also shown.

### 4.1. SAD phasing of leucine-rich repeat protein   

#### 4.1.1. Sample description and data collection   

Crystals of the leucine-rich repeat (LRR) protein from *L. interrogans* containing residues 30–377 were kindly provided by Ahmed Haouz (Institut Pasteur) and William Shepard (Synchrotron SOLEIL). Details of the crystal preparation have been published elsewhere (Miras *et al.*, 2015 ▸).

Data collection from crystals of LRR was carried out on beamline I04 at Diamond Light Source, UK using 1% transmission and an exposure time of 0.04 s per image. A total of 1027 images, comprising a 154° scan, with a rotation per image of 0.15°, were measured using an X-ray wavelength of 1.2 Å, which is just shorter than the Zn *K* absorption edge. The data are available at https://doi.org/10.5281/zenodo.1048928.

#### 4.1.2. Data processing   

The data were processed with *xia*2 (Winter, 2010 ▸) using *DIALS* for indexing, refinement and integration using *POINTLESS* (Evans, 2006 ▸) and *AIMLESS* (Evans & Murshudov, 2013 ▸) for scaling. Anomalous pairs were separated in scaling and merging, with the resolution limit estimated automatically by *xia*2 as 1.45 Å (based on CC_1/2_ > 0.5 after the first cycle of scaling); the overall merging statistics are shown in Table 1 ▸. While the *R*_meas_ value in the outer shell may appear excessive (in excess of 100%), the half sets of data are still significantly correlated, with CC_1/2_ = 0.669 (Karplus & Diederichs, 2012 ▸), and thus contribute usefully to the data set.

#### 4.1.3. Phasing   

Structure solution was carried out using the anomalous signal from native Zn^2+^ ions, estimated to have d*I*/σ(d*I*) ≃ 1.29, with the *SHELXC*/*D*/*E* pipeline (Sheldrick, 2010 ▸). The resolution cutoff for substructure determination was 2.5 Å. *SHELXD* found eight heavy-atom sites with occupancy greater than 25%, with CC_all_ of 40.38% and CC_weak_ of 21.39%. *SHELXE* was able to trace the backbone of the protein successfully in the original hand, with a CC of 44.73% (*versus* 8.83% for the inverse), clearly identifying the true solution. Density-modified phases were used for automated model building with *Buccaneer* (Cowtan, 2006 ▸) and a single molecule per asymmetric unit was built, resulting in an initial *R*_work_ of 26.36% and *R*_free_ of 28.37% before further refinement.

#### 4.1.4. Refinement and model completion   

**Figure 2:**
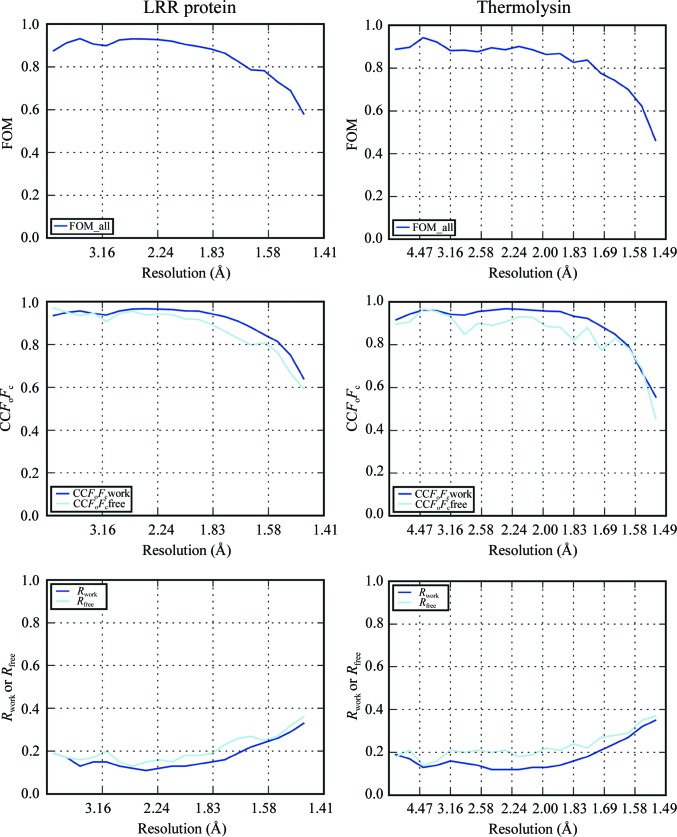
Refinement statistics [mean of cosine of phase error (FOM), CC between *F*_o_ and *F*_c_, and *R* factors] for LRR and thermolysin against data processed with *xia*2 and *DIALS*. While there are no surprises for LRR, for thermolysin it is important to note that the statistics remain well behaved as the *I*/σ(*I*) of the data tends towards zero.

Statistics for the refinement are shown in Table 1 ▸. All residues from the expression construct were built, as well as several ligands from the crystallization condition and 402 water molecules. Statistics of the final refinement run are presented in Fig. 2 ▸, with the figure of merit (FOM), the correlation coefficient of the difference map (CC*F*_o_ *F*_c_), *R*_work_ and *R*_free_ plotted against resolution.

### 4.2. Molecular replacement of thermolysin with weak data   

#### 4.2.1. Sample description and data collection   

Crystals of thermolysin were produced from commercially sourced thermolysin from *Bacillus thermoproteolyticus* (Calbiochem). The protein was dissolved in 100 m*M* MES pH 6.0, 45%(*v*/*v*) DMSO to a final concentration of 100 mg ml^−1^ by gently shaking the mixture at room temperature for 1 h. To remove aggregates and other particles, the mixture was centrifuged for 10 min at 15 000*g* and 4°C. Equal amounts of protein solution and a well solution consisting of 50 m*M* MES pH 6.0, 1 *M* sodium chloride and 45%(*v*/*v*) DMSO were mixed as a sitting drop and equilibrated over a reservoir solution consisting of 35%(*v*/*v*) saturated ammonium sulfate at a temperature of 20°C. Crystals with space group *P*6_1_22 and unit-cell parameters *a* = *b* = 92.35, *c* = 127.71 Å formed within a few days.

Data were collected on beamline I03 at Diamond Light Source following a low-dose, high-multiplicity strategy: 0.05% X-ray beam transmission and 0.1 s per 0.1°, generating a total of 7200 images, *i.e.* two full rotations, using an X-ray wavelength of 1.2 Å. This resulted in data with around 200 000 total counts per image, or an average number of counts per pixel of 0.03. The data are available at https://doi.org/10.5281/zenodo.49559.

#### 4.2.2. Data processing   

Data were processed with *xia*2 as for the previous example in §[Sec sec4.1.2]4.1.2, although a resolution limit of 1.5 Å was explicitly set to test the behaviour of the software in the asymptotic limit, *i.e.* where 〈*I*/σ(*I*)〉 tends to 0. Statistics are reported in Table 1 ▸. The data have an overall 〈*I*/σ(*I*)〉 of 13.3, whereas in the high-resolution shell it drops to near 0. The *R*_meas_ values of 22.6% for the data overall and 26.20% in the outer shell reflect the very low photon counts; however, the data half sets (*i.e.* CC_1/2_) are still significantly correlated (25.8%) in the outer shell as the overall multiplicity of the data exceeds 70.

#### 4.2.3. Phasing   

Phases were determined by molecular replacement with *Phaser* (McCoy *et al.*, 2007 ▸) using PDB entry 2tlx (English *et al.*, 1999 ▸) as the search model with all water molecules and ligands removed. The phasing was straightforward, with a TFZ score of >8, an LLG of >160, a refined LLG of 8684 and one molecule in the asymmetric unit.

#### 4.2.4. Refinement   

For refinement a free set of 2500 reflections (5% of the total) was used. Final *R*-value statistics of *R*_work_ = 15.7% and *R*_free_ = 20.5% were obtained, with the values for the highest resolution shell being 35.2% and 36.8%, respectively. 302 water molecules and additional ligands from the crystallization condition, as well as a short peptide in the active site, were built.

#### 4.2.5. Paired refinement   

Following the protocol of Karplus & Diederichs (2012 ▸), the thermolysin structure was refined with data from 1.8 to 1.5 Å resolution in steps of 0.01 Å, *i.e.* 31 refinement runs. The atomic positions were first perturbed by an average of 0.25 Å with *phenix.pdbtools* (Adams *et al.*, 2010 ▸), after which the refinement was performed with data to the defined resolution limit. *R*_work_ and *R*_free_ were then computed using data to 1.8 Å resolution.

**Figure 3:**
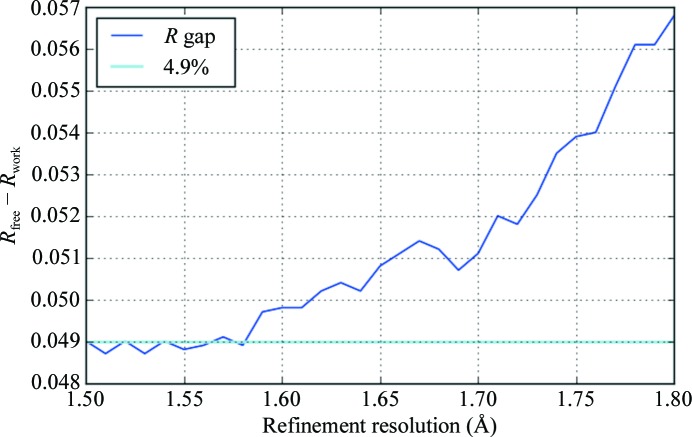
*R*-factor gap using data to 1.8 Å resolution as a function of the resolution of the data used for the paired refinement. There is a clear reduction in the difference between *R* and *R*_free_ using the weaker measurements to around 1.56 Å resolution.

Perturbation of the atoms was sufficient to increase the *R* factor from around 14 to 18% overall for the 1.6 Å resolution data, after which the residuals settled to their previous values. As may be seen in Fig. 3 ▸, there is a measurable improvement in the gap between *R* and *R*_free_ calculated to 1.8 Å resolution using data to around 1.56 Å resolution. Beyond this point (*i.e.* from 1.50 to 1.56 Å) both the *R*_work_ and *R*_free_ to 1.8 Å resolution do not change substantially, suggesting that this is the true resolution limit of the data. It is, however, helpful to note that the additional measurements beyond this limit did no apparent harm to the structure refinement.

### 4.3. Chemical crystallography   

**Figure 4:**
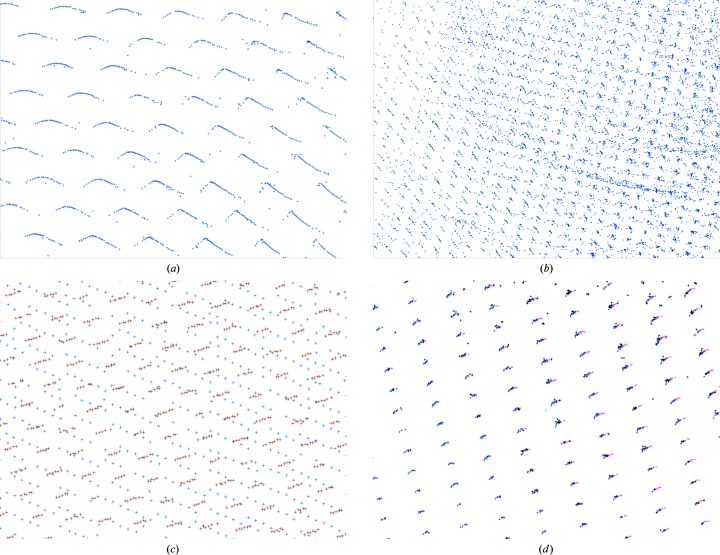
Commonly encountered reciprocal-space pathologies using *dials.reciprocal_lattice_viewer*. (*a*) Problems with image headers, such as an incorrect beam centre or an inverted rotation axis, may lead to an apparent distortion in the lattice. Depending on the severity of the distortion, autoindexing may identify an incorrect lattice or result in an offset in the assigned Miller indices. (*b*) Visible features that are not part of the primary lattice, such as points arranged in a spherical shell, may indicate the presence of ice rings or low-quality powder samples. (*c*) Split crystals or multiple lattices are visible as a set of two or more intersecting lattices. Unindexed reflections and reflections identified as belonging to distinct lattices are coloured separately to aid visualization. (*d*) Multiple sweeps from a single crystal on a multi-axis goniometer can be combined for display, with each sweep uniquely coloured.

Whilst MX is the dominant application of crystallography at third-generation synchrotron sources, Diamond Light Source has a dedicated facility for chemical crystallography at beamline I19. Mathematically, the analysis process is identical to MX; however, there are a few practical differences. Firstly, the geometry of the experiment tends to be more complex, with 2θ offsets routinely applied to the detector and multi-axis goniometers in use for the majority of experiments. Secondly, the volume of the unit cell is typically smaller, resulting in fewer observed reflections despite diffraction to higher resolution. To address these challenges in *xia*2 the default behaviour for small-molecule data is to simultaneously index reflection data from all sweeps, relying on the accurate mapping to reciprocal space shown in Fig. 4 ▸(*d*). Finally, the normal operating energy of the beamline is around 19 keV, compared with MX beamlines which typically operate around 8–13 keV. This last factor substantially affects the operating efficiency of the PILATUS 2M, as the probability of recording a photon at 19 keV with a 320 µm thick sensor can be as low as 36%.

The data set used as an example here was collected from l-cysteine, and the data are available online at https://doi.org/10.5281/zenodo.51405. The data consist of four sweeps: a 180° φ scan at 2θ = 0° followed by three 170° ω scans at φ = 0, 120 and 240°, with 2θ = 30° on a fixed-χ (χ = 57.74°) goniometer. The data processed with *xia*2 gave the merging statistics in Table 2 ▸. Structure solution with *SHELXT* (Sheldrick, 2015 ▸) was straightforward and refinement with *OLEX*2 (Dolomanov *et al.*, 2009 ▸) gave a final *R*_1_ of 3.04% (details are given in Table 2 ▸).

A particular concern for chemical crystallography is the greater dynamic range in intensities, particularly for centric space groups that give rise to more extreme intensity distributions. The use of photon-counting detectors, however, means that good results have been achieved with data recorded in a single sweep, where the reflection intensities span 3–4 orders of magnitude. Since *DIALS* uses both summation and profile-fitting integration methods, the option in *AIMLESS* to use an intensity-weighted combination of these was used, such that the stronger reflections are dominated by summation-integrated values and the weaker reflections by the results of profile fitting.

## 5. Diagnostic tools   

While the main focus of *DIALS* is the implementation of new software the integration of X-ray diffraction data, diagnostic tools have also been developed, which help the user to understand the behaviour of the *DIALS* algorithms in more detail. In addition, at each stage of the analysis presented previously, reports are available to assess the quality of the results.

### 5.1. Image viewer   

*DIALS* provides an image viewer based on previous work (Sauter *et al.*, 2013 ▸) that can be used to inspect diffraction images and diagnose issues with data processing. The viewer can also display the location of reflections from spot finding or integration, including the shoebox regions, and has the option to sum a number of consecutive images together for display; this can be especially useful for viewing weak, sparse or fine-sliced data in order to provide an interpretable diffraction pattern. *Appendix B* [App appb] includes example usage of the *DIALS* image viewer command line and other diagnostic tools described below.

Additionally, the image viewer can be used to optimize the parameters affecting spot finding: the effect of changing the spot-finding parameters can be observed by displaying the threshold view of the image. This may be useful when commissioning a new type of detector or experiment.

### 5.2. Reciprocal lattice viewer   

In many cases the failure point in processing a diffraction data set is in indexing. While the algorithms used in *DIALS* (Steller *et al.*, 1997 ▸; Sauter *et al.*, 2004 ▸; Bricogne, 1986*a* ▸; Gildea *et al.*, 2014 ▸) are generally robust, if they fail to index the reflections the program may offer little insight into the underlying cause, for example an incorrect description of the experimental geometry. In some cases, overlaying the found spot positions over the images may provide an indication of the cause of indexing failure, but a particularly powerful diagnostic tool is to view their positions in reciprocal space using the *DIALS* reciprocal lattice viewer. In common with other tools such as *RLATT* (Bruker AXS Inc., Madison, Wisconsin, USA) and *EwaldPro* (Rigaku Oxford Diffraction, Oxford, England), the ability to visualize the results of spot finding in reciprocal space allows the immediate diagnosis of many indexing problems. Fig. 4 ▸ demonstrates some of the most common phenomena that are observed. In case of incorrectly defined geometry the parameters may be adjusted within the GUI, allowing common causes of failure to be easily corrected. This is valuable when commissioning a new beamline, where an accurate description of the geometry may not be available.

### 5.3. Crystal health   

**Figure 5:**
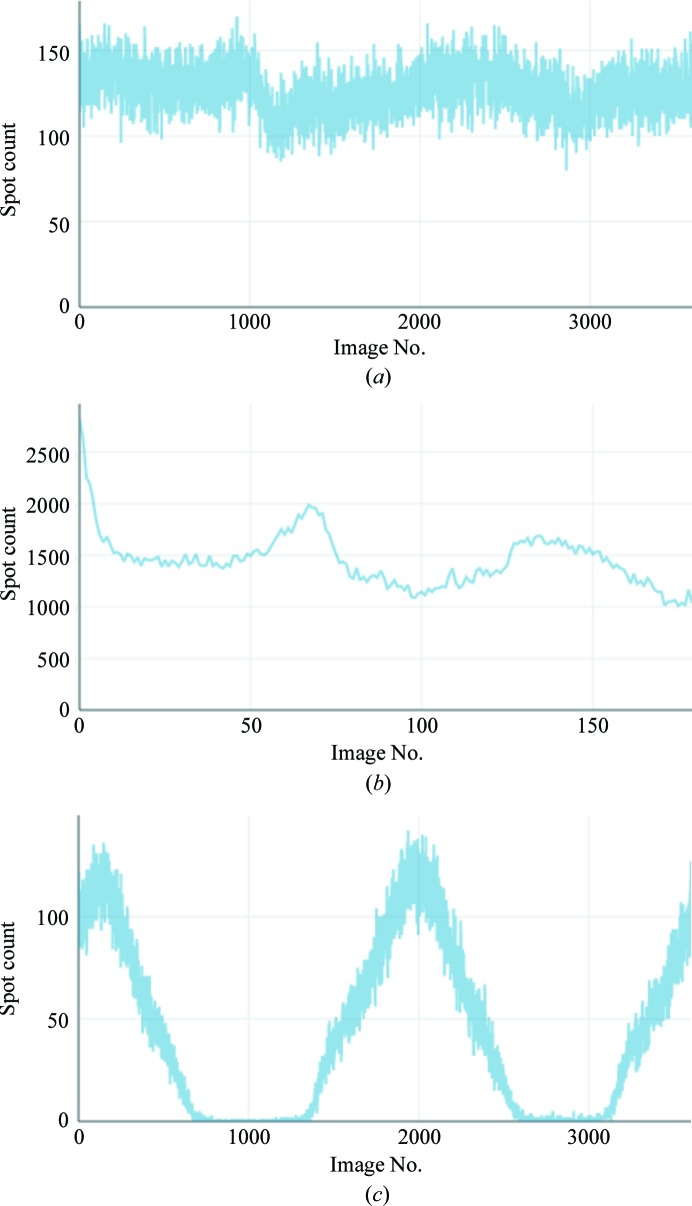
Spot count per image plots generated by the *dials.report* tool for three data sets. (*a*) shows what may be expected when there is no substantial radiation damage, (*b*) when there is substantial radiation damage and (*c*) when a poorly centred sample is rotated out of the beam for part of the scan. These indicators may very rapidly be used to diagnose issues with data sets without needing to individually inspect the images.

Prior to the arrival of pixel-array detectors it was possible to inspect every image as it was collected. When data sets consist of many thousands of finely sliced images recorded at a rate greater than ten per second, manual inspection becomes impractical, leading to a loss of insight into the evolution of the sample, and issues such as radiation damage or sample misalignment may be overlooked. Within *DIALS*, spot-finding results can be used to overcome this loss of insight through a summary of the number of spots found on every image: if there is no substantial radiation damage and the diffraction is approximately isotropic this may be expected to be approximately constant, as shown in Fig. 5 ▸(*a*), or to vary sinusoidally with a period of 180°. If a crystal has suffered severe radiation damage (Fig. 5 ▸ *b*) then the number of spots will typically decrease systematically, while sample-centring issues (Fig. 5 ▸ *c*) may result in clearly visible ‘blank’ regions. In many cases, ‘problem’ data sets may be identified at this stage prior to any thorough analysis of the data. This is used at Diamond Light Source to provide rapid feedback to users (Winter & McAuley, 2011 ▸).

### 5.4. *DIALS* report   

**Figure 6:**
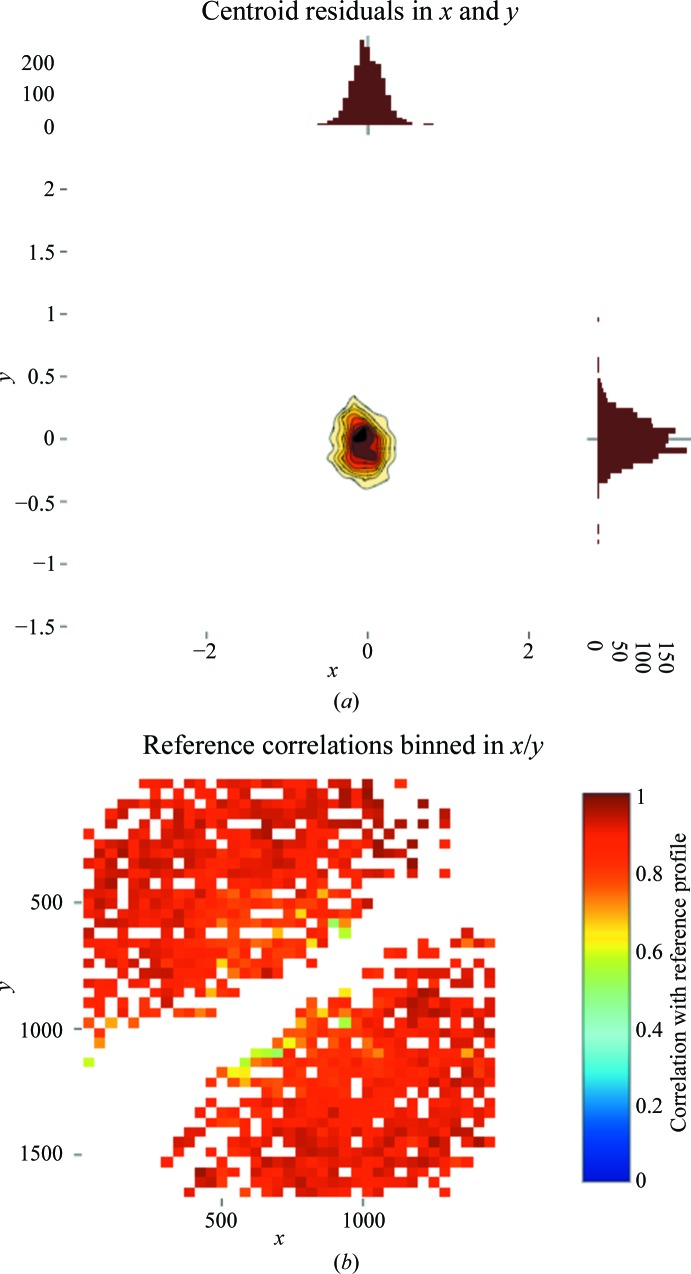
Images generated by *dials.report* showing (*a*) a histogram of *x*, *y* deviations between observed and calculated spot positions from refinement and (*b*) correlation between modelled and observed spot profiles in integration. The diagonal blank region corresponds to reflections close to the rotation axis in a φ scan (both taken from the small-molecule example in the main text).

The output of each analysis step is typically a list of reflections and a description of the current state of the experimental model. The *dials.report* tool takes the information contained in these files and generates HTML reports containing critical diagnostic results such as histograms of the deviation between observed and predicted reflections (Fig. 6 ▸ *a*) and correlations between the model and observed reflection profiles (Fig. 6 ▸ *b*).

## 6. Conclusions   

The *DIALS* project, comprising the framework and some key algorithms, is presented together with results of its application to good-quality data measured at Diamond Light Source. The *DIALS* project set out to develop (i) a framework for the implementation of novel algorithms for data integration, (ii) a toolbox of algorithms and (iii) user-facing tools for the processing of X-ray diffraction data. As illustrated here, these goals have been met and *DIALS* has now been released. In writing the *DIALS* software, the authors have aimed to provide the community with an open-source platform for further algorithm development as well as a suite of tools to enable data processing. To date (17 September 2017) the software has been cited in 92 PDB depositions.

*DIALS* has already been used to process data at X-ray free-electron laser sources (Brewster *et al.*, 2016 ▸; Lyubimov *et al.*, 2016 ▸; Young *et al.*, 2016 ▸). Future developments in *DIALS* will include its extension for use with other sources and methods, including electron diffraction.

*DIALS* is available for download from https://dials.github.io and is distributed with the *CCP*4 (Winn *et al.*, 2011 ▸) and *PHENIX* (Adams *et al.*, 2010 ▸) software packages.

## Supplementary Material

Data from crystal of Leucine-Rich Repeat Protein from Leptospira interrogans recorded during routine commissioning on Diamond Light Source beamline I04 URL: https://doi.org/10.5281/zenodo.1048928

Low dose, high multiplicity thermolysin X-ray diffraction data from Diamond Light Source beamline I03 URL: https://doi.org/10.5281/zenodo.49559

L Cysteine Data collected 05/03/2016 at Diamond Light Source I19-1 URL: https://doi.org/10.5281/zenodo.51405

## Figures and Tables

**Table 1:** Crystallographic parameters, data, phasing and refinement statistics Values in parentheses are for the highest resolution shell.

	LRR	Thermolysin
Crystal parameters		
Space group	*P*4_2_2_1_2	*P*6_1_22
Unit-cell parameters (Å)	*a* = *b* = 121.49738 (10), *c* = 57.02179 (9)	*a* = *b* = 92.35414 (9), *c* = 127.7128 (2)
Data statistics		
Resolution range (Å)	57.03–1.45 (1.48–1.45)	79.97–1.50 (1.54–1.50)
No. of unique reflections	75939 (3767)	50728 (3264)
Multiplicity	10.6 (8.8)	73.3 (52.5)
*R*_merge_	0.077 (1.114)	0.222 (24.722)
*R*_meas_	0.081 (1.183)	0.226 (26.202)
*R*_p.i.m._	0.025 (0.391)	0.026 (3.495)
Completeness (%)	100.0 (100.0)	97.8 (86.8)
〈*I*/σ(*I*)〉	14.7 (1.7)	13.3 (0.1)
CC_1/2_	0.999 (0.669)	1.000 (0.258)
Wilson plot *B* factor (Å^2^)	14.13	16.09
Phasing		
*SHELXD*		
CC_all_ (%)	40.38	
CC_weak_ (%)	21.39	
No. of sites with occupancy > 25%	8	
*SHELXE*		
CC, original hand (%)	44.73	
CC, inverse hand (%)	8.83	
Refinement		
Resolution (Å)	54.40–1.45 (1.48–1.45)	79.97–1.50 (1.54–1.50)
No. of reflections		
Total	75880 (5255)	50699 (3064)
Working set	72061 (4986)	48191 (2932)
Free set	3722 (269)	2500 (132)
*R*_work_	0.152 (0.335)	0.157 (0.352)
*R*_free_	0.187 (0.367)	0.205 (0.368)
No. of non-H atoms		
Protein	2899	2456
Waters	402	302
Zn^2+^ ions	9	2
Cl^−^ ions	—	2
Ca^2+^ ions	—	4
Peptide	—	18
Acetate	16	8
Tris	8	—
2-Propanol	8	—
DMSO	—	4
Sulfate	—	5
R.m.s. deviations from ideal		
Bond lengths (Å)	0.009	0.010
Bond angles (°)	1.376	1.332
Ramachandran plot (%)		
Preferred regions	94.65	96.25
Allowed regions	5.35	3.75
Outliers	0.0	0.0

**Table 2:** Merging statistics for L-cysteine data obtained on Diamond Light Source beamline I19-1

Crystal parameters	
Space group	*P*2_1_2_1_2_1_
Unit-cell parameters (Å)	*a* = 5.4278 (9), *b* = 8.1444 (13), *c* = 12.0391 (21)
*V* (Å^3^)	532.2007 (955)
Formula	C_3_H_7_NO_2_S
Data statistics	
Resolution range (Å)	6.74–0.58 (0.63–0.58)
No. of unique reflections	1552 (263)
Multiplicity	7.6 (3.7)
*R*_merge_	0.033 (0.068)
*R*_meas_	0.035 (0.078)
*R*_p.i.m._	0.011 (0.036)
Completeness (%)	95.0 (83.3)
〈*I*/σ(*I*)〉	28.5 (13.5)
CC_1/2_	1.000 (0.992)
Refinement	
*R*[*F* ^2^ > 2σ(*F* ^2^)]	0.0304
*R* (all data)	0.0312
*wR*(*F* ^2^) (all data)	0.0887
Goodness-of-fit (*F* ^2^)	1.103
Flack parameter	0.05 (3)
No. of reflections	7388
No. of parameters	66
No. of restraints	0

## Appendix A Parallax correction

The physics of direct-conversion pixel-array detectors, particularly those with a silicon sensor, gives rise to a small distortion of the diffraction image: the diffraction spots are elongated owing to the passage of the photons through the sensor. This gives rise to a predictable effect on the central impact (Duisenberg *et al.*, 2003 ▸) of the reflection, which may be corrected by the ‘pixel-to-millimetres’ mapping.

The absorption of photons in a material is given by the Beer–Lambert law. Specifically, the fraction of photons transmitted a distance *x* into a material with linear attenuation coefficient μ is given by

From this, it can be shown that for a sample of thickness *t*, the attenuation length *L*_a_, the distance into the sample at which the mean absorption occurs, is

For a diffracted beam vector **s**_1_ striking a detector with normal vector  and thickness *t*_0_, the effective distance *t* = *t*_0_/(**s**_1_ · ). Therefore,

The offset for a predicted ray impinging on the detector with fast axis **e** *_x_* and slow axis **e** *_y_* is then

## Appendix B Command lines

The *DIALS* distribution includes a number of tools which were first implemented for debugging but were later found to be more generally useful: examples of the output of these have been included in the main text. In general, the tools take an experiment model file and optionally a spot list.

View diffraction images optionally with overlay of strong spot positions, optionally summing images for viewing very finely sliced data.

View a projection of the reciprocal lattice either from ‘raw’ diffraction centroids or indexed reflections (Fig. 4 ▸). Both the experimental geometry and reflection data are needed.

Generate a report from the *DIALS* analysis, the contents of which will depend on the stage in the analysis. This generates an HTML report dials-report.html (Fig. 6 ▸).
